# The Role of CD44 and ERM Proteins in Expression and Functionality of P-glycoprotein in Breast Cancer Cells

**DOI:** 10.3390/molecules21030290

**Published:** 2016-03-01

**Authors:** Deep Pokharel, Matthew P. Padula, Jamie F. Lu, Ritu Jaiswal, Steven P. Djordjevic, Mary Bebawy

**Affiliations:** 1 Discipline of Pharmacy, The Graduate School of Health, University of Technology Sydney, Sydney NSW 2007, Australia; deep.pokharel@student.uts.edu.au (D.P.); Jamie.f.lu@student.uts.edu.au (J.F.L.); ritu.jaiswal@uts.edu.au (R.J.); 2 Proteomics Core Facility, University of Technology Sydney, Sydney NSW 2007, Australia; matthew.padula@uts.edu.au; 3 The ithree Institute, University of Technology Sydney, Sydney NSW 2007, Australia; steven.djordjevic@uts.edu.au

**Keywords:** CD44, Ezrin-Radixin-Moesin, extracellular vesicles, multidrug resistance, P-glycoprotein

## Abstract

Multidrug resistance (MDR) is often attributed to the over-expression of P-glycoprotein (P-gp), which prevents the accumulation of anticancer drugs within cells by virtue of its active drug efflux capacity. We have previously described the intercellular transfer of P-gp via extracellular vesicles (EVs) and proposed the involvement of a unique protein complex in regulating this process. In this paper, we investigate the role of these mediators in the regulation of P-gp functionality and hence the acquisition of MDR following cell to cell transfer. By sequentially silencing the FERM domain-binding proteins, Ezrin, Radixin and Moesin (ERM), as well as CD44, which we also report a selective packaging in breast cancer derived EVs, we have established a role for these proteins, in particular Radixin and CD44, in influencing the P-gp-mediated MDR in whole cells. We also report for the first time the role of ERM proteins in the vesicular transfer of functional P-gp. Specifically, we demonstrate that intercellular membrane insertion is dependent on Ezrin and Moesin, whilst P-gp functionality is governed by the integrity of all ERM proteins in the recipient cell. This study identifies these candidate proteins as potential new therapeutic targets in circumventing MDR clinically.

## 1. Introduction

Extracellular vesicles (EVs) comprise one of the many modes of cell-to-cell communication in both prokaryotic and eukaryotic organisms [1,2]. They serve as vehicles for the exchange of proteins and nucleic acids, and have been shown to confer cancer traits, including multidrug resistance (MDR) and an enhanced metastatic capacity, in cancer cells [1,3,4,5,6,7]. MDR arises from the ability of cancer cells to resist the cytotoxic effects of chemotherapeutics by exporting them out from the cytosol and membrane to the extracellular space [8,9]. ATP-binding cassette (ABC) transporter proteins, including P-glycoprotein (P-gp) and MRP-1, are two transporters whose overexpression in cancer cells confers MDR [2,10].

Previously, we have described the transfer of functional MDR proteins via EVs, which are released by the ubiquitous cellular phenomenon of membrane budding [3,4]. EVs derived from drug-resistant cancer cells transfer these integral plasma membrane proteins to recipient drug-sensitive cells, effectively conferring functional MDR within a matter of hours [3,4]. Our previous proteomic analysis of EVs derived from resistant breast cancer cells identified candidate mediators unique to these cells relative to their drug sensitive counterparts [1]. Specifically, we showed the selective packaging of Ezrin-Radixin-Moesin (ERM) and CD44, among other proteins, in EVs shed from drug-resistant breast cancer cells [1]. We proposed that this protein complex may play a role in the intercellular transfer of P-gp and in conferring MDR.

ERM refers to a family of closely related FERM domain proteins (F stands for 4.1 protein) that are required to interface membrane proteins with the cytoskeleton [11]. The subcellular localisation and functionality of P-gp is dependent on the cytoskeleton and cytoskeletal binding proteins [11]. Indeed, P-gp co-localises with Ezrin in IFN-γ-treated monocyte derived macrophages and with ERM proteins on pseudopods/uropods in drug-resistant lymphoid cells [11,12]. Furthermore, an actin-P-gp interaction is required for the endosomal trafficking of P-gp to the plasma membrane [13]. Likewise, disruption of the ERM/P-gp association impairs P-gp function and results in a cellular redistribution of P-gp [11,14].

CD44 is an adhesion molecule that belongs to the hyaluronan (HA) receptor family of cell-surface glycoproteins [15]. CD44 has been shown to initiate the metastatic spread of tumor cells and is involved in cell adhesion and motility [16]. P-gp and CD44 are co-regulated, co-immunoprecipitated, and their interaction promotes cell migration and invasion in cancer [17]. Interestingly, ERM proteins also bind the C-terminal domain of actin filaments with CD44, demonstrating the essential role of direct (physical) or indirect (functional) interaction of proteins at every level of cellular function [18,19,20].

In this study, we use proteomic profiling and comparative analysis to expand on our previous studies and validate the molecular mediators unique to the drug-resistant cancer state that governs P-gp functionality in whole cells and the intercellular transfer of functional P-gp via membrane vesicles. We demonstrate that ERM are important mediators common to the acquisition of intercellular MDR in both resistant leukemic and breast cancer cell models. We also again observe a selective packaging of CD44 in resistant breast cancer-derived EVs consistent with our previous findings [1,15]. Furthermore, we examine the role of this complex in regulating P-gp expression and function in both breast cancer cells that are inherently multidrug-resistant as well as those cells that have acquired resistance through intercellular transfer. We show that, Radixin and CD44 are required for regulating P-gp drug efflux in resistant breast cancer cells at a functional level. Similarly, the vesicular transfer of P-gp via EVs is highly dependent on the presence of Ezrin, Radixin and Moesin in the recipient drug-sensitive host cells. These findings have significant implications for understanding the molecular regulators governing P-gp transfer and functionality conferred through vesicular cell-to-cell communication. This knowledge will aid in identifying novel therapeutic targets for the circumvention of MDR.

## 2. Results

### 2.1. EV Isolation and Validation

**Figure 1:**
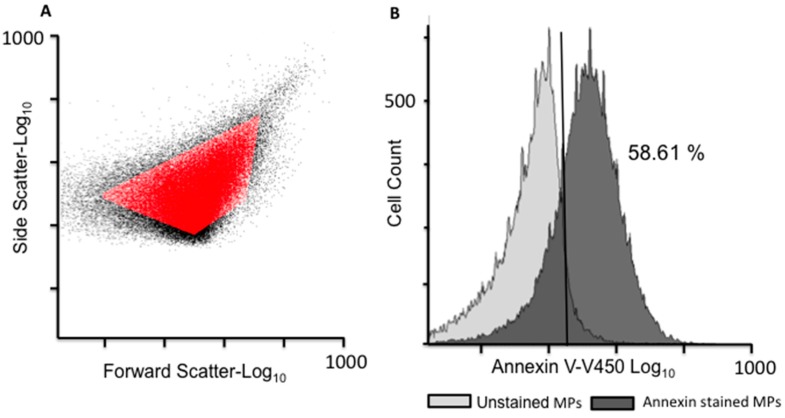
EVs (extracellular vesicles) isolated from VLB_100_ cells. (**A**) EVs isolated from VLB_100_ cells were analysed via flow cytometry (FCM) and defined as elements smaller in diameter than 1 μm. Latex beads of 0.3–1.1 µM (Sigma, Castle Hill, Australia) were used to define the upper limit of the forward scatter. The threshold was set on the side scatter to prevent exclusion of the smallest events. (**B**) 58.61% of the gated EV population was positive for Annexin V-V450. Data is representative of a typical experiment (n = 3).

The EVs used in this study were derived from a drug-sensitive human acute lymphoblastic leukaemia cell line (designated CEM for simplicity) and its drug resistant sub-clone VLB_100_. Similarly, the drug-sensitive breast cancer cells (MCF-7) and their MDR variant (MCF-7/Dx, frequently named as Dx cells for simplicity) were also used. Dx-cells and VLB_100_-cells are MDR variants of MCF-7 and CEM cells, respectively, and are the result of the prolonged and incremental exposure to sub-lethal concentrations of anticancer drugs [3,15]. EVs were isolated from confluent cells by differential centrifugation, as described previously [3,5,15,21]. Flow cytometry **(**FCM) (LSRII, BD Biosciences, San Jose, CA, USA) was used to validate the isolated EV fraction for size and surface expression of phosphatidylserine (PS), as described previously [3,4,21]. The EV population was defined as gated events between 0.3–1.1 µm in diameter as determined using latex beads. Of the gated vesicle population, 58.61% were positive for Annexin V-V450 (560506; BD Biosciences) staining, confirming the presence of PS (Figure 1), which is consistent with our previous reports [3,4,21].

### 2.2. Proteomic Profiling by LC/MS/MS and Western Blotting

**Figure 2:**
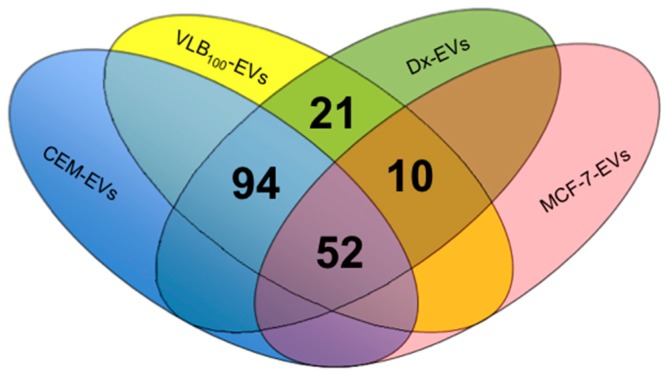
Proteomic profiling by LC/MS/MS. EVs derived from leukemic cells were compared to the proteome of resistant breast cancer-derived EVs [1]. LC-MS/MS identified a total of 589 EV proteins with 177 proteins common to both the MDR breast cancer EVs (Dx-EVs) and MDR leukemic EVs (VLB_100_-EVs). Included here were P-gp, Ezrin, Radixin and Moesin.

We have applied the shotgun proteomic approach to phenotype EVs derived from leukemic cells and compared these to the breast cancer-derived proteome previously reported by us [1]. A total of 589 EV-derived proteins were identified by LC-MS/MS and Scaffold (v4.4.5) from breast cancer and leukaemia cell lines (Supplementary Table). Of the 589 proteins identified, 177 were common to the resistant state being present in both drug-resistant-derived VLB_100_-EVs and Dx-EVs (Figure 2).

Our previous studies in Dx-EVs showed a selective packaging of Ezrin, Radixin, Moesin and CD44 in resistant breast cancer cell-derived EVs [1]. Again, in resistant leukemic-derived EVs, we detected the presence of Ezrin, Radixin, Moesin and P-gp, consistent with our previous study [1]. However, unlike our previous findings, CD44 was not present in resistant leukaemia-derived EVs but only in sensitive EVs. The 177 proteins were further classified based on gene ontology and pathway analysis using the PANTHER classification system [22]. Twenty-six proteins were reported to play a role in cell communication, 37 in cell localization and six in cell adhesion. Sixteen proteins were identified as transmembrane transporters and 22 proteins had a role as cytoskeletal components. Several proteins were found to play a significant role in cancer pathways, including the 14-3-3 protein sigma in the p53 pathway, the 14-3-3 protein zeta/delta in the PI3 kinase pathway, and ras-related C3 botulinum toxin substrate 1 in the VEGF-signalling pathway. Using the String 10 database, proteins such as solute carrier family 16 and topoisomerase A were shown to associate directly with P-gp/*ABCB1* (Supplementary Figure S1). 

Given the role of the FERM domain-binding proteins and CD44 in P-gp membrane localisation in whole cells and their presence in EVs derived from resistant cells, we examined the role of these proteins in regulating P-gp functionality in resistant breast cancer cells as well as in the intercellular transfer of functional P-gp by microvesicles. We validated our LC-MS/MS data for presence of ERM and CD44 using Western blot analysis. Ezrin, Radixin, and Moesin were detected around 80 kDa in both CEM and VLB_100_ cells and in their EVs; however, these proteins were in greater abundance in the EVs relative to their corresponding cells (Supplementary Figure). Although we detected CD44 in CEM-EVs by LC-MS/MS, we failed to detect CD44 by Western blot using the monoclonal antibody (clone EPR1013Y; Abcam), which is consistent with our previous findings [15]. In using an anti-CD44 polyclonal antibody (HPA005785; Sigma-Aldrich) we were unable to again detect CD44 in VLB_100_-EVs, despite being able to detect multiple bands in CEM and VLB_100_ cells, and CEM-EVs only (Supplementary Figure S2).

### 2.3. Gene Silencing of ERM and CD44 Affects P-gp Drug Efflux in Resistant Breast Cancer Cells

**Figure 3:**
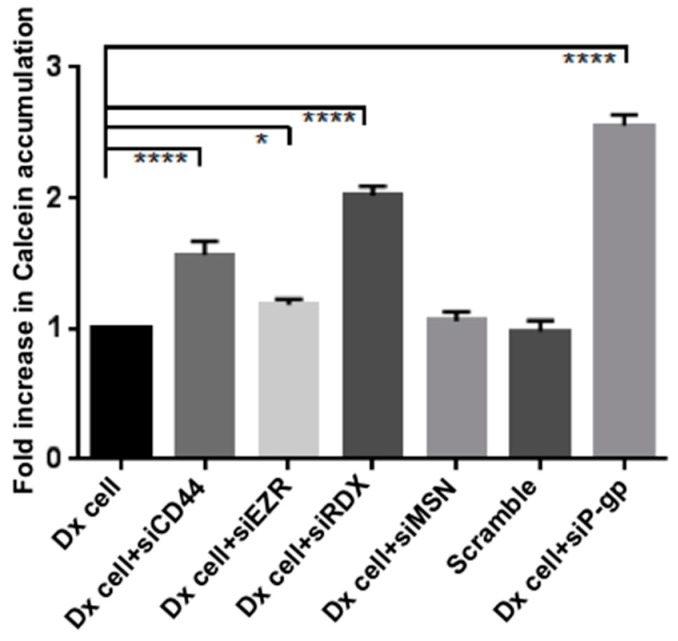
Gene silencing of ERM and CD44—Effect on drug efflux in MCF-7/Dx cells. Dx-cells were silenced with siRNA targeting CD44 (siCD44), Ezrin (siEZR), Radixin (siRDX), Moesin (siMSN) and P-gp (siP-gp) over three days and drug efflux evaluated using FCM. Data represents mean ± SD (n = 3). * *p* < 0.05, **** *p* < 0.0001.

We used siRNA silencing of Ezrin, Radixin, Moesin and CD44 to evaluate the role of each protein in regulating P-gp function in drug-resistant breast cancer cells. P-gp functionality was assessed using the Calcein dye exclusion assay as previously described by us [4]. We silenced each protein over a three-day period to ensure capturing the protein half-life and allowing for sufficient time for the decay of any endogenous protein present [23,24] (Supplementary Figure S3). Dx cells displayed a 2.56 ± 0.08 fold increases in Calcein accumulation after silencing with P-gp siRNA (designated siP-gp for simplicity). The silencing of CD44 (siCD44) and Radixin (siRDX) also resulted in a significant increase in Calcein accumulation by 1.57 ± 0.10 and 2.02 ± 0.07 folds, respectively, demonstrating a role for CD44 and Radixin in regulating P-gp drug efflux. In contrast, silencing of Ezrin (siEZR) and Moesin (siMSN) resulted in an insignificant increase in Calcein accumulation of 1.18 ± 0.05 and 1.06 ± 0.07 fold, respectively (Figure 3). Verapamil hydrochloride, which is a classical inhibitor of P-gp [5], was also used as a control (Supplementary Figure S4). In presence of 60 μM of verapamil, we see an increase in accumulation across all conditions resulting from inhibition of P-gp.

**Figure 4:**
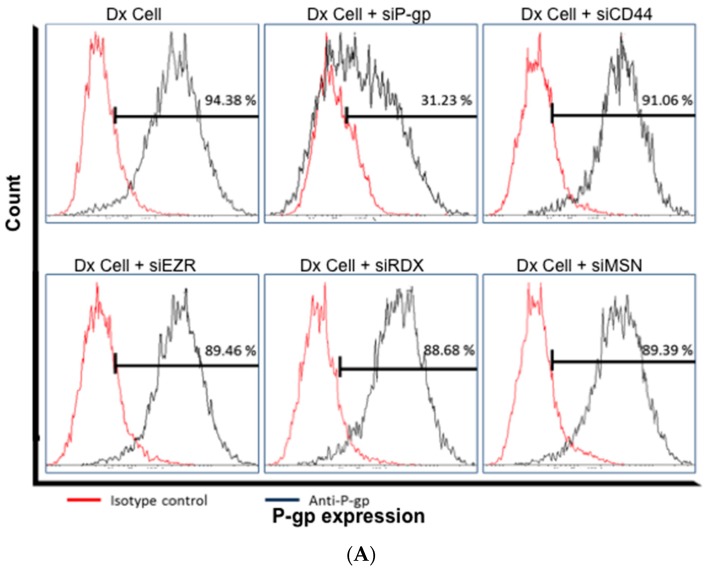

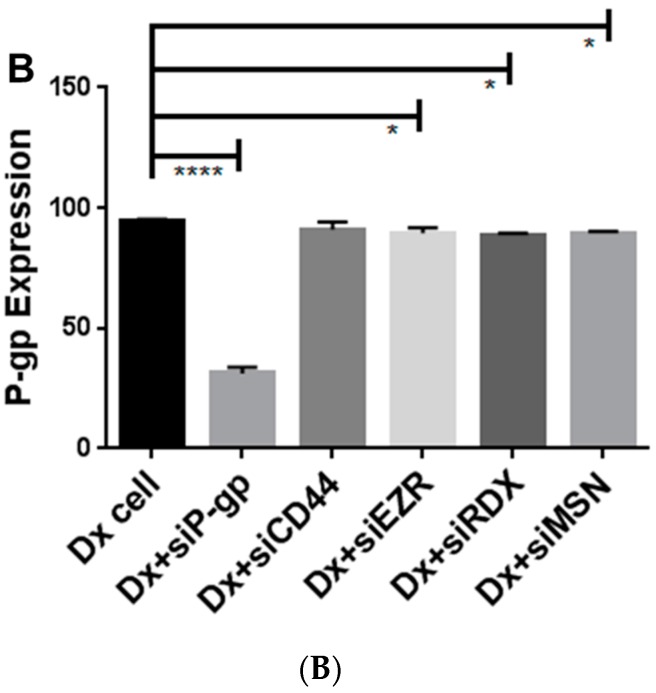
(**A**) Histogram and (**B**) Graph, representing the gene silencing of ERM and CD44—effect on P-gp expression in MCF-7/Dx cells: Dx-cells were silenced with siRNA targeting CD44 (siCD44), Ezrin (siEZR), Radixin (siRDX), Moesin (siMSN) and P-gp (siP-gp) over three days and P-gp expression was evaluated using FCM. Data represents mean ± SD (n = 3).

Additionally, Ezrin, Radixin, Moesin and CD44 in Dx cells were silenced to assess their contribution in regulating the cellular expression of P-gp. We observed no effect on P-gp expression following silencing of these proteins as measured by FCM (Figure 4).

### 2.4. Gene Silencing of P-gp Has no Effect on ERM and CD44 Expression in Resistant Breast Cancer Cells

**Figure 5:**
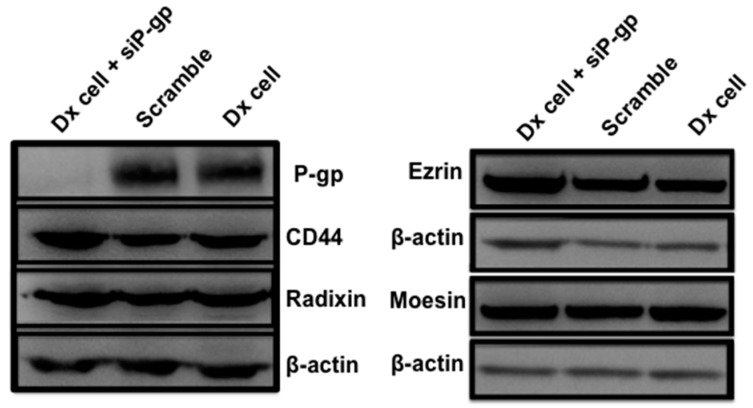
Western blot analysis of gene silencing—Effect of P-gp on expression of ERM and CD44 in Dx-cells. Dx-cells were silenced with siRNA targeting P-gp over three days. The effect of P-gp (170 kDa) on the expression of Ezrin (~80 kDa), Radixin (~80 kDa), Moesin (~80 kDa) and CD44 (82 kDa) was evaluated using Western blot. β-actin was used as the loading control. Data is representative of a typical experiments (n = 3).

Similarly, knockdown of P-gp had no effect on the expression of Ezrin, Radixin, Moesin or CD44 (Figure 5).

### 2.5. Role of ERM in the Vesicular Transfer of P-gp to Recipient Drug Sensitive Breast Cancer Cells

**Figure 6:**
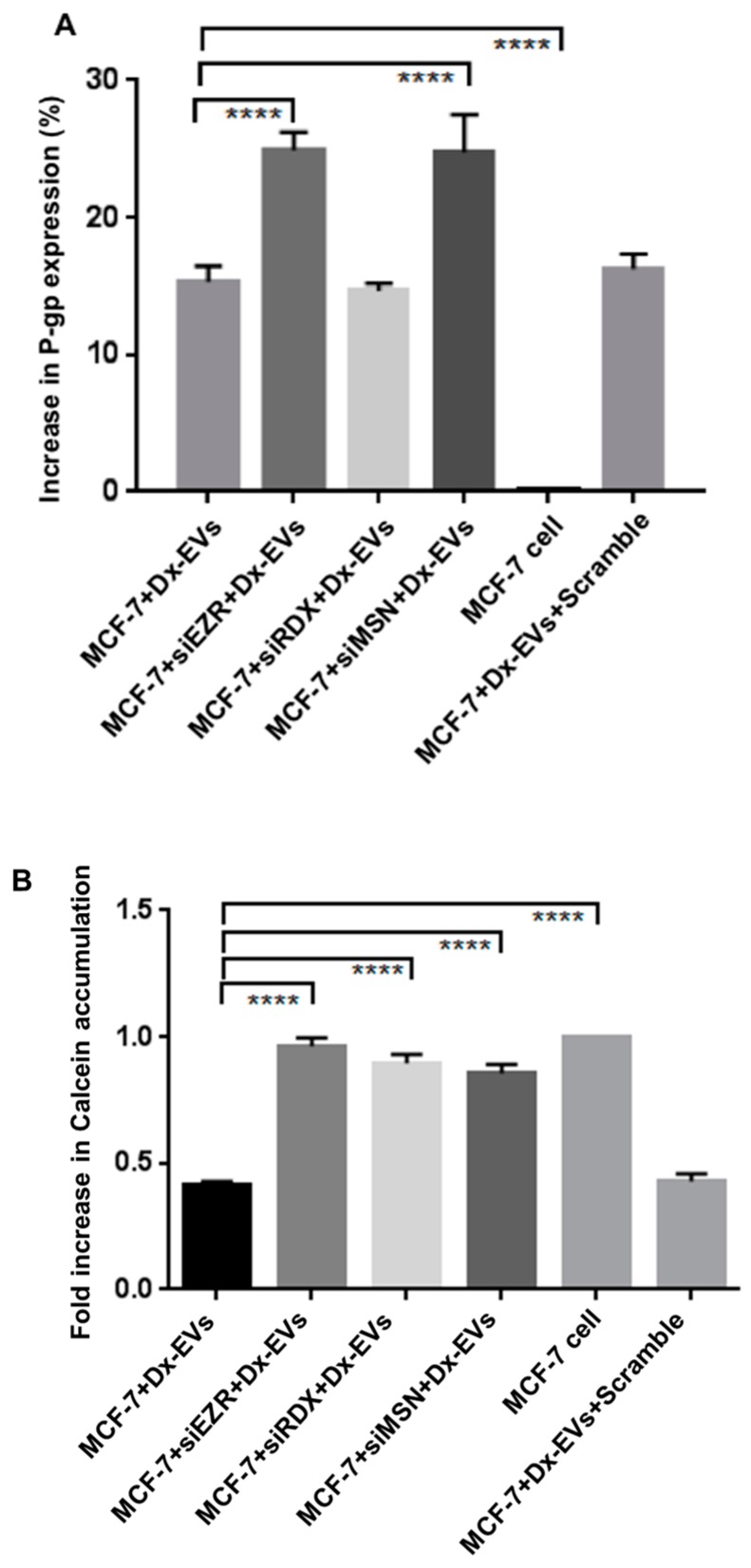
Effect of gene silencing in the recipient cell on vesicular transfer of P-gp and drug efflux. MCF-7 cells were silenced with siRNA targeting, Ezrin (siEZR), Radixin (siRDX), Moesin (siMSN), and following which cells were co-cultured with MCF-7/Dx-EVs for 4 h.; (**A**) P-gp expression and (**B**) Calcein-AM uptake was evaluated by FCM. Data represents mean ± SD (n = 3). **** *p* < 0.0001.

To verify the role of ERM proteins in the intercellular transfer and function of P-gp we used siRNA to silence Ezrin, Radixin and Moesin in drug sensitive recipient cells and co-cultured these with Dx-EVs to assess whether these proteins were required for P-gp transfer and membrane insertion. We did not silence CD44 in these recipient cells, as the MCF-7 cells do not express CD44, as demonstrated in our previous work [1]. We observed that 15.32% ± 1.15% of MCF-7 cells were positive for P-gp after co-culturing with Dx-EVs, which is consistent with our previous findings [3,5]. We observed no significant difference in the extent of P-gp transfer upon silencing of Radixin, with 14.64% ± 0.58% of the population detecting positive for P-gp (Figure 6A). However, we observed a significant increase in P-gp transfer within the population up to 24.90% ± 1.33% and 24.79% ± 1.59% in MCF-7 cells with prior siEZR and siMSN treatment of the recipient cell, respectively (Figure 6A, *p* < 0.0001). Upon co-culture of MCF-7 cells with Dx-EVs, we observed a significant 2.4 fold decrease (*p* < 0.0001) in Calcein accumulation following EV co-culture relative to accumulation in MCF-7 cells alone. Despite the higher P-gp expression, we observed a modest change in Calcein accumulation in recipient cells after siEZR, siRDX and siMSN and EV co-culture, respectively (Figure 6B), emphasizing the role of ERM, in particular with P-gp drug efflux.

## 3. Discussion

The overexpression of the multidrug transporter, P-gp by cancer cells is a major obstacle to the successful treatment of cancer. In MDR cells, P-gp is reported to resist the cytotoxic effect of chemotherapeutics by exporting them from the cytosol to the extracellular space [25]. Current pharmacological strategies aimed at circumventing MDR clinically are compromised by a lack of specificity, dose-limiting toxicity and a lack of efficacy [26]. Hence alternative strategies are sought to overcome MDR in cancer therapy.

Recent evidence has shown that the mechanism of drug efflux may not only depend on the level of P-gp expression but also on the protein-protein interactions shared between P-gp and other proteins. For example, interactions between plasma membrane and cytoskeletal proteins are reported to play a significant role in drug efflux, as well as membrane trafficking, signal transduction and various other cellular functions [11,27]. In particular, MDR pumps are mostly localized in the polarized sites of epithelial cells and the polarized distribution of P-gp depends on the arrangement of cytoskeletal proteins such as actin and ERM [28,29]. Similarly, the interaction between CD44 and hyaluronic acid (HA) also influences the expression of P-gp. CD44 is a single chain, transmembrane glycoprotein that includes a N-terminal domain, distal-membrane domain and a hyaluronan-binding domain [30]. Binding of HA to CD44 has been shown to stimulate multidrug and metabolic transporters that are important in cancer drug resistance [30]. P-gp, ERM, CD44 and actin are all co-localized in MDR cells and they may directly interact with each other to affect drug efflux [11,31,32]. Briefly, this interaction occurs with the binding of ERM proteins directly to the C-terminal domain of actin filaments [33] and to the cytoplasmic NH_2_-terminal domain of CD44 [19], whereas P-gp binds to the amino acid residues 149–242 of the N-terminal domain of Ezrin [14].

Our previous studies in EVs shed from multidrug resistant breast cancer cells showed a selective packaging of Ezrin, Radixin, Moesin and CD44 in resistant breast cancer cell-derived EVs [1]. Using proteomic profiling we identified 177 proteins common to the resistant state being present in both drug-resistant-derived VLB_100_-EVs and Dx-EVs. Amongst these proteins we again detected Ezrin, Radixin and Moesin. Consistent with our previous findings, however, CD44 was only found to be selectively packaged in the Dx-EVs [1]. An interesting phenomenon that we were the first to report on in the context of EV-mediated P-gp transfer was ‘tissue selectivity’ [15]. We showed that the vesicular transfer of P-gp by MDR breast cancer-derived EVs displays a degree of tissue selectivity in contrast to MDR leukemic-EVs [15]. In defining the molecular basis governing this tissue selectivity, we identified the selective packaging of CD44 in MDR breast cancer-derived EVs [1,15]. The presence of CD44 only in resistant breast cancer cell-derived EVs and not in resistant leukemic-derived EVs may relate to this phenomenon. This, however, remains to be examined. Collectively, our findings demonstrate the presence of these proteins in resistant breast cancer EVs and resistant leukemic EVs and may propose a role in regulating the transfer and insertion of P-gp into recipient cells and /or be the consequence of a selective packaging mechanism common to both types of drug-induced cancer cells. We further examined the role of these proteins in affecting P-gp expression and function in Dx cells and in affecting P-gp transfers to recipient cells through their vesicles. 

In this study, we provide evidence that in Dx cells, P-gp function is mediated by ERM and CD44 proteins. We performed gene-silencing experiments in Dx cells and showed that the cells with the full complex of ERM, CD44 and P-gp demonstrated the lowest calcein accumulation of calcein, consistent with the active functionality of P-gp. Calcein-AM is a non-fluorescent membrane-permeable drug [4]. Upon entering the cells, calcein-AM is cleaved by endogenous esterases to its fluorescent non-membrane-permeable form, calcein, which remains trapped inside the cell. Although ERM proteins share high sequence identity (~75%), [11] our results show significant differences in their roles in regulating drug efflux. The siRDX cells, in particular, resulted in a significant fold increase of 2.05 (*p* < 0.0001) in calcein accumulation relative to scrambled siRNA controls (Figure 3). siCD44 cells also resulted in a significant fold more calcein accumulation of 1.60 (*p* < 0.0001) relative to the controls. Similar results were shown by Cain *et al.*, using siRNA knockdown of CD44 which resulted in an increased sensitivity to doxorubicin [34]. Others have also shown that the association between hyaluronic acid-CD44 with receptor kinases and transporter proteins play a significant role in drug resistance and malignancy [16,35]. Collectively, these findings suggest that P-gp drug efflux is dependent on the presence of CD44 as well as Radixin. We observed no significant effect on P-gp expression upon silencing any of these proteins using FCM (Figure 4), hence suggesting their role in the structural stabilisation of P-gp only, rather than in regulating protein expression. 

Moreover, to determine if ERM proteins mediate the vesicular transfer of P-gp and acquisition of MDR, we performed silencing experiments in the host MCF-7 cells and co-cultured them with Dx-EVs. Consistent with our previous work, we observed the transfer of P-gp in 4 h [3]. In siRDX cells we observed no increase in P-gp transference. Surprisingly, we observed an increase in transfer of P-gp from Dx-EVs to the drug-sensitive recipient cells, even after prior siEZR and siMSN treatment. The reason as to why the silencing of Ezrin and Moesin resulted in greater transfer of P-gp to recipient cells via EVs is currently unknown. As membrane localization is required for P-gp functionality [36], the silencing of the Ezrin and Moesin proteins may affect cell and cytoskeletal ultrastructure in a manner conducible for vesicular membrane fusion and transfer of protein. We observed an increase in Calcein accumulation following the silencing of all ERM proteins, suggesting that P-gp functionality is dependent on their presence and regulates the membrane insertion of P-gp in recipient cells during vesicular transfer (Figure 6). This is the first report to demonstrate that the intercellular transfer of P-gp in the recipient cell membrane is dependent on Ezrin and Moesin. However, the functionality acquired through this process is dependent on the integrity of all ERM members.

## 4. Materials and Methods

### 4.1. Cell Culture

The drug-sensitive human acute lymphoblastic cells CCRF-CEM (CEM), their MDR variant VLB_100_ cells, the drug sensitive breast cancer cells and their MDR variant Dx cells were used in these studies. The cell lines have been validated as appropriate models for the study of P-gp-mediated MDR *in vitro* and *in vivo* [3,4,8,9]. Cells were cultured in RPMI 1640 medium (Sigma-Aldrich, Castle Hill, Australia), supplemented with 10% (*v/v*) heat-inactivated fetal bovine serum (Life Technologies, Mulgrave, Australia) and maintained in the absence of antibiotics at 37 °C and 5% CO_2_.

### 4.2. Harvesting EVs

We have previously described the isolation of EVs and their validation for morphology, size and resistance protein expression [4,5,15]. The procedure for isolation of EVs was as previously described [3]. In brief, upon reaching confluence, the cells and supernatant were harvested and centrifuged for 5 min at 500 *g* to pellet cells and cell debris. The supernatant was again centrifuged for 5 min at 500 *g* to further clarify the supernatant. Following this, the supernatant was centrifuged for 1 h at 15,000 *g* at 17 °C and the pellet-containing EVs were washed in serum-free media and further centrifuged for 2 min at 2000 *g* to remove debris. The supernatant was further centrifuged for 30 min at 18,000 *g* at 17 °C to pellet the EVs. The EV pellet was resuspended in serum-free RPMI-1640 and the fraction validated for size, phosphatidylserine exposure and P-gp expression by flow cytometry (FCM) (LSRII, BD Biosciences), as described in [4,8,9]. 

### 4.3. Protein Extraction

The EV pellet was resuspended in 1 mL of alkaline extraction reagent (pH 8.8) containing 7 M urea, 2 M thiourea, 50 mM tris(hydroxymethyl)methylamine-HCl and 1% C7BzO, and was sonicated for 30 s at 40% power, four times using a high intensity ultrasonic processor (50 watt, Sonics and Materials Inc, Newtown, CT, USA). The samples were reduced and alkylated in the alkaline protein solution by adding tributylphosphine solution to a final concentration of 5 mM and acrylamide monomers to a final concentration of 20 mM. The reaction was quenched by adding 1 M dithiothreitol solution (final concentration 20 mM) and centrifuged at 17,000 *g* for 5 min to pellet any insoluble material. Finally the protein was precipitated with 5 volumes of acetone before resuspension in 1% SDS (Sodium Dodecyl Sulfate). Total protein concentration was determined using a Qubit™ fluorometer (Invitrogen, Mount Waverley, Australia) *as per* the manufacturer’s protocol.

### 4.4. SDS-PAGE and Trypsin In-Gel Digestion

One hundred micrograms of extracted EV protein was mixed with 2 × SDS sample buffer containing Tris-HCl, glycerol, SDS, bromophenol blue and water (pH 8.8) at a ratio of 2:1 (protein:SDS buffer), and sonicated in a water bath for 5 min followed by centrifugation for 5 min at 16,500 *g* at ambient temperature. Proteins were separated using 4%–10% SDS-PAGE at a voltage of 150 V in MES SDS running buffer (Invitrogen, Life Technologies). The gel was fixed with 40% Methanol/10% Acetic acid for 30 min with gentle shaking before staining with Coomassie blue overnight. In-gel trypsin digestion was performed as previously described [1,37]. Briefly, the gel was sectioned according the MW markers and the sections diced into 1 × 1 mm pieces which were de-stained by adding 50% acetonitrile (ACN)/50 mM NH_4_HCO_3_ and incubated for 10 min at ambient temperature. The process was repeated until the stain disappeared. 100% ACN was added to dehydrate the gel pieces before rehydration with 12.5 ng/µL of trypsin solution (Trypsin gold-MS grade, Promega) and overnight incubation at 37 °C. After incubation, the supernatant was collected after sonication in a water bath for 10 min, followed by another sonication following the addition of 30 µL of 50% ACN/2% formic acid. The solution was added to the previously collected peptides and the volume reduced to 15 µL by rotary evaporation. The peptide solution was centrifuged at 14,000 *g* for 10 min to remove any insoluble material prior to LC/MS/MS analysis.

### 4.5. LC/MS/MS

Using an Eksigent AS-1 autosampler connected to a Tempo nanoLC system (Eksigent, Redwood City, CA, USA), 10 µL of the sample was loaded at 20 µL/min with MS-loading solvent (2% ACN + 0.2% Trifluoroacetic Acid) onto a C8 trap column (CapTrap. Michrom Biosciences, Auburn, CA, USA). After washing the trap for three minutes, the peptides were washed off the trap at 300 nL/min with MS solvent A (2% ACN + 0.2% Formic Acid) onto a PicoFrit column (75 mm × 150 mm, New Objective) packed with Magic C18AQ resin (Michrom Biosciences). Peptides were eluted from the column and into the source of a QSTAR Elite hybrid quadrupole time-of-flight mass spectrometer (ABSciex) using the following program: 5%–50% MS solvent B (98% ACN + 0.2% Formic Acid) over 30 min, 50%–80% MS solvent B over 5 min, 80% MS solvent B for 2 min, 80%–5% for 3 min. The eluting peptides were ionised from the PicoFrit column at 2300 V. An Intelligent Data Acquisition [38] experiment was performed, with a mass range of 350–1500 Da continuously scanned for peptides of charge state 2+–5+ with an intensity of more than 30 counts/s. Selected peptides were fragmented and the product ion fragment masses measured over a mass range of 100–1500 Da. The mass of the precursor peptide was then excluded for 15 s.

### 4.6. Data Analysis

Three biological replicates of total EV proteins were analysed by LC/MS/MS. The data files produced by QSTAR Elite were searched using Mascot Daemon (version 2.4 provided by the Walter and Eliza Hall Institute) [39] and searched against the LudwigNR database (comprised of the UniProt, plasmoDB and Ensembl databases (vQ213)) with the following parameter settings: Taxonomy: Homo sapiens; Fixed modifications: none; Variable modifications: propionamide, oxidised methionine and deamidated asparagine; Enzyme: semi-trypsin; Number of allowed missed cleavages: 3; Peptide mass tolerance: 100 ppm; MS/MS mass tolerance: 0.2 Da; Charge state: 2+, 3+ and 4+. Scaffold (v4.4.5, Proteome Software, Portland, OR, USA) was used to validate and compare MS/MS-based peptide and protein identifications, as previously described [1]. Peptide identifications were accepted if they could be established at greater than 95.0% probability as specified by the Peptide Prophet algorithm [40]. Protein identifications were accepted if they could be established at greater than 95.0% probability and contained at least 2 identified peptides in 2 replicates. Protein probabilities were assigned by the Protein Prophet algorithm [41]. Proteins that contained similar peptides and could not be differentiated based on MS/MS analysis alone were clustered by Scaffold to satisfy the principles of parsimony. For FCM, data was analysed using one-way analysis of variance (ANOVA). *p*-values of <0.05 were accepted as being statistically significant. 

### 4.7. *In Vitro* Silencing

Transfection of siRNA (QIAGEN) was performed on Dx-cells for 3 days. Cells were plated 24 h before transfection in 96 well plates with RPMI 1640 medium (Sigma-Aldrich) supplemented with 10% (*v/v*) heat-inactivated foetal bovine serum (Life Technologies) and maintained in the absence of antibiotics under 37 °C and 5% CO_2_. The cells were transfected with a final concentration of 50 nM of siRNA, purchased from QIAGEN, targeting P-gp; 5′-ATCGAGTCACTGCCTAATAAA-3′, CD44; 5′-CTGAAATTAGGGCCCAATTAA-3′, Ezrin; 5′-CTCGGCATTATTCTCGAATCA-3′, Radixin; 5′-ATGGTGGACCCTACTATTCAT-3′, and Moesin; 5′-CAGGATGTCAACTGACCTAAA-3′, using Lipofectamine 2000 (Invitrogen) *as per* the manufacturer’s recommendation. The cells were transfected once more after 24 h and harvested the following day. Scrambled siRNA and untreated cells were used as relevant controls.

### 4.8. Calcein-AM Dye Exclusion Assay

Calcein-AM was used to examine effects on P-gp-mediated drug efflux, which has been extensively used to measure P-gp functionality [4,5]. Briefly, the siRNA-silenced cells were harvested and washed twice with complete culture medium and exposure to 0.1 μM of Calcein-AM for 1 h at 37 °C and 5% CO_2_. The cells were subsequently washed three times with PBS and re-suspended in 200 μL PBS for FCM analysis.

### 4.9. Western Blotting

Twenty micrograms of total cellular and EV lysates were separated by SDS-PAGE before transferring to PVDF membrane (Pall Australia, VIC, Australia), as described in [4]. The membrane was blocked overnight with 5% skim milk in PBS and 0.05% Tween 20 and then incubated with anti-P-gp mAb (clone F4; Sigma-Aldrich), anti-Ezrin mAb (clone 3C12; Life Science), anti-Moesin mAb (clone 38/87; Sigma-Aldrich), anti-Radixin pAb (SAB2500859; Sigma-Aldrich) or anti-CD44 pAb (HPA005785; Sigma-Aldrich) for 1 h. Anti-β-actin (clone AC-74; Sigma-Aldrich) was used as the internal control, followed by 1 h incubation with anti-Mouse HRP secondary antibody (Promega, NSW, Australia) at a 1:10,000 dilution. Protein expression was visualised using the ECL (enhanced chemiluminescence) system (Roche Applied Science, Castle Hill, NSW, Australia). The membranes were imaged using the luminescent image analyser LAS-3000 (Fujifilms, Brookvale, NSW, Australia).

### 4.10. Vesicular Transfer Experiments

In a 96-well U bottom culture plate, 100 µg of Dx-EVs and VLB_100_-EVs were co-cultured with MCF-7 and CEM cells, respectively, for 4 h in a total of 200 μL complete culture medium at 37 °C and 5% CO_2_, as described in [3,4]. Unbound EVs were removed by centrifuging at 500 *g* for 5 min after 4 h and washed twice with PBS.

### 4.11. Statistical Analysis

All statistical analyses were performed using GraphPad Prism version 5.0 for Windows Software (GraphPad Software, La Jolla, CA, USA). Gene silencing and functional measurements were expressed as means ± SD of three independent experiments. The data was analysed using one-way analysis of variance (ANOVA) and *p*-values of <0.05 were accepted as being statistically significant. 

## 5. Conclusions

EVs from drug-resistant cells package extracellular/intracellular transporter proteins, adhesion proteins, cytoskeletal proteins, actin-binding proteins and, particularly, cytoskeletal-binding proteins, that assist in conferring MDR in recipient cells. Our findings suggest the presence of an interactive network of proteins namely, ERM and CD44 with P-gp, in breast cancer cells. It further indicates that despite the cancer cells displaying similar or higher amounts of P-gp, disruption of this interaction complex leads to the loss of P-gp drug efflux functionality. Defining this interactive complex of ERM and CD44 provides opportunities for targeted therapies aimed at counteracting cancer MDR clinically. This study also furthers our knowledge on the intercellular mechanisms governing the transfer and acquisition of functional P-gp in cancer.

## Supplementary Materials

The following Table: “Proteome profile of EVs derived from leukaemia cell lines” and “Supplementary Figure” is available online at http://www.mdpi.com/1420-3049/21/3/290/s1.

## Abbreviations

The following abbreviations are used in this manuscript:

ERM: Ezrin-Radixin-Moesin
EVs: Extracellular vesicles
MDR: Multidrug resistance
P-gp: P-glycoprotein
