# Supplementary material for: The Role of CD44 and ERM Proteins in Expression and Functionality of P-glycoprotein in Breast Cancer Cells
Source: Molecules. 2016 Mar 1;21(3):290. doi: 10.3390/molecules21030290 (PMC6273996; doi:10.3390/molecules21030290)
Supplement: Supplementary file 1 [file molecules-21-00290-s001.zip › Supplementary Figure.pptx]

## Slide 1
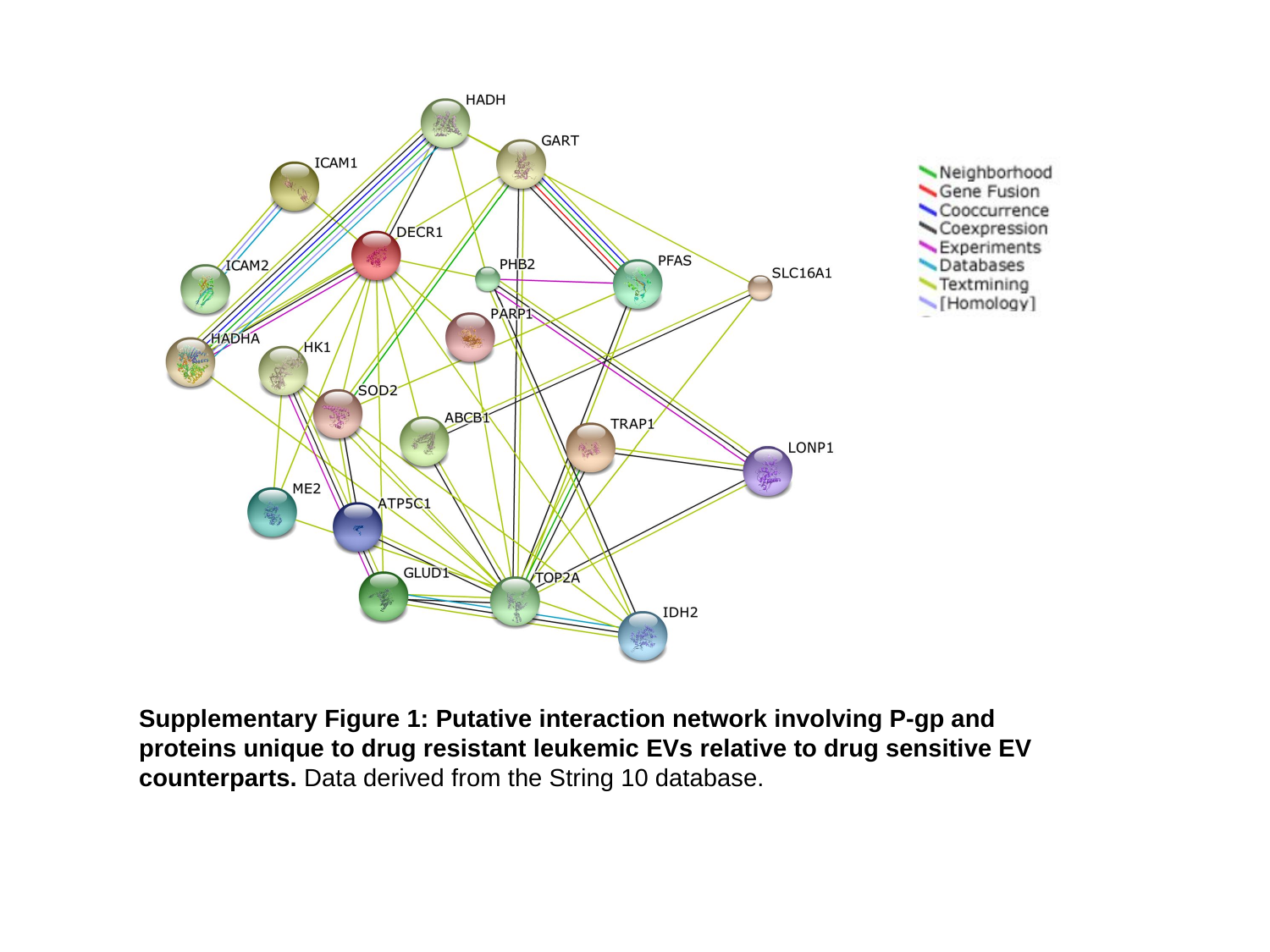

Supplementary Figure 1: Putative interaction network involving P-gp and proteins unique to drug resistant leukemic EVs relative to drug sensitive EV counterparts. Data derived from the String 10 database.

## Slide 2
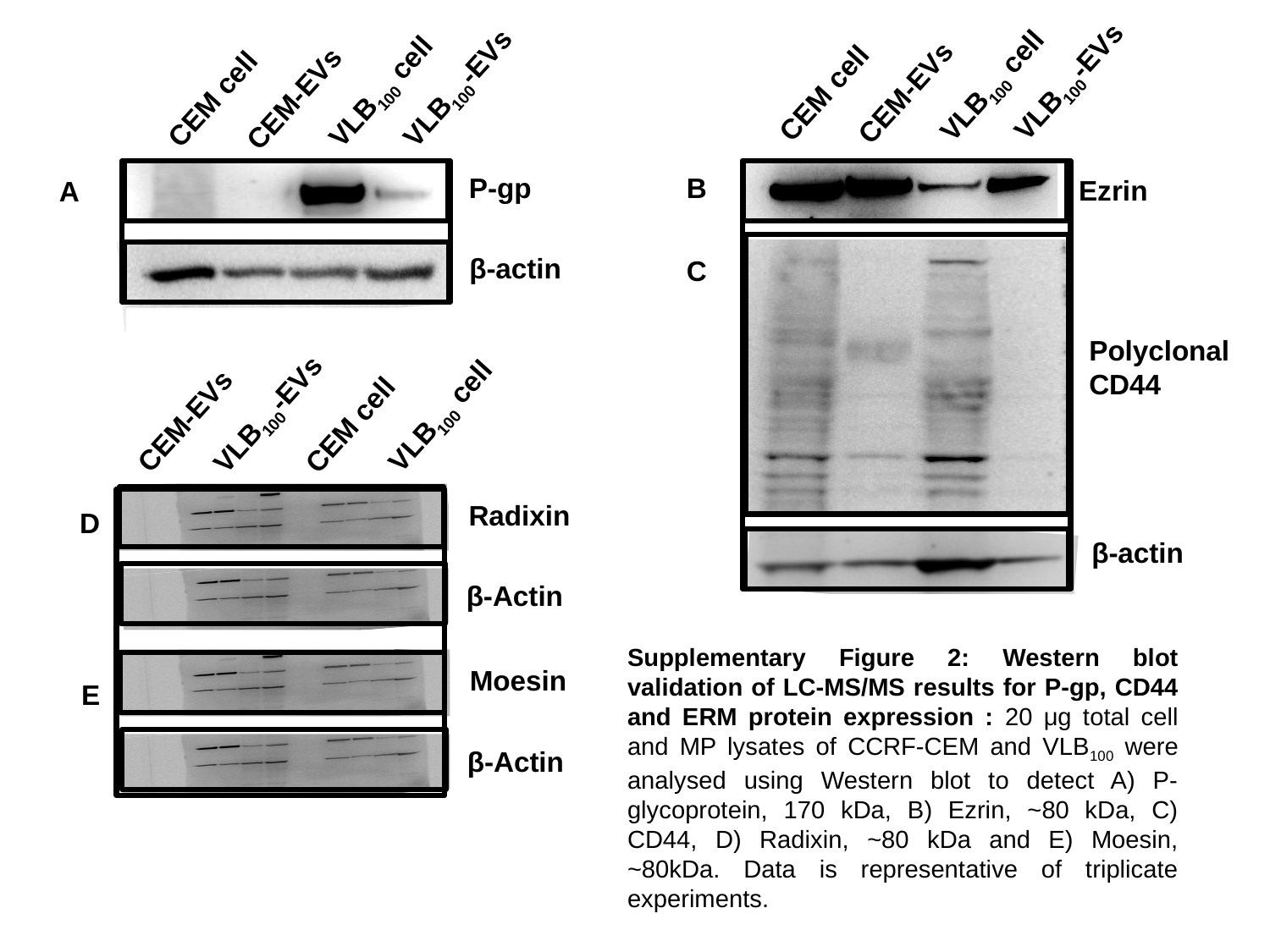

VLB100-EVs
VLB100 cell
VLB100-EVs
VLB100 cell
CEM cell
CEM-EVs
CEM cell
CEM-EVs
P-gp
B
Ezrin
A
β-actin
C
VLB100-EVs
VLB100 cell
CEM-EVs
CEM cell
Radixin
β-Actin
Polyclonal
CD44
D
β-actin
Moesin
β-Actin
Supplementary Figure 2: Western blot validation of LC-MS/MS results for P-gp, CD44 and ERM protein expression : 20 μg total cell and MP lysates of CCRF-CEM and VLB100 were analysed using Western blot to detect A) P-glycoprotein, 170 kDa, B) Ezrin, ~80 kDa, C) CD44, D) Radixin, ~80 kDa and E) Moesin, ~80kDa. Data is representative of triplicate experiments.
E

## Slide 3
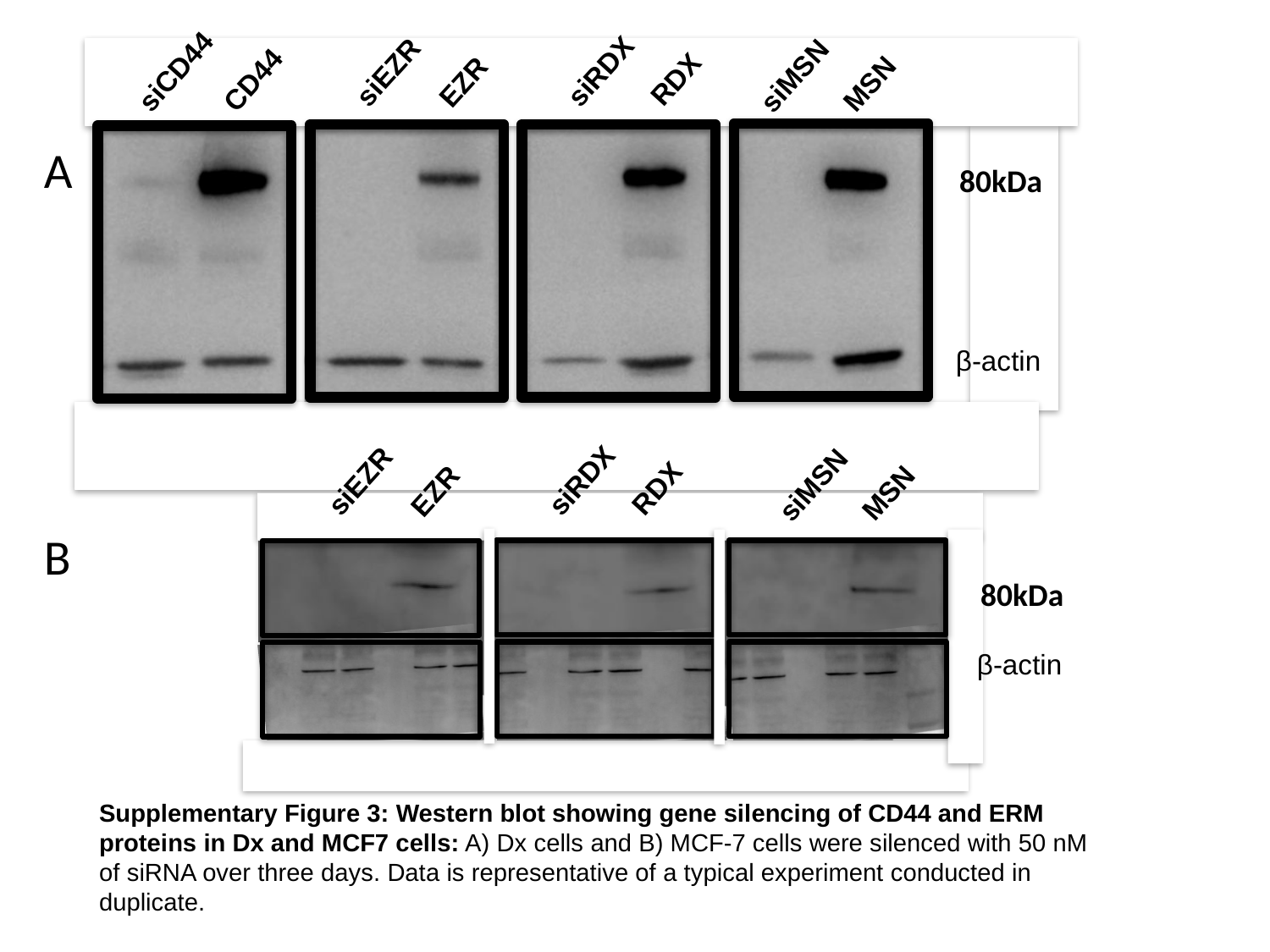

siRDX
siCD44
siEZR
siMSN
RDX
CD44
EZR
MSN
A
80kDa
β-actin
siRDX
siEZR
siMSN
RDX
EZR
MSN
B
80kDa
β-actin
Supplementary Figure 3: Western blot showing gene silencing of CD44 and ERM proteins in Dx and MCF7 cells: A) Dx cells and B) MCF-7 cells were silenced with 50 nM of siRNA over three days. Data is representative of a typical experiment conducted in duplicate.

## Slide 4
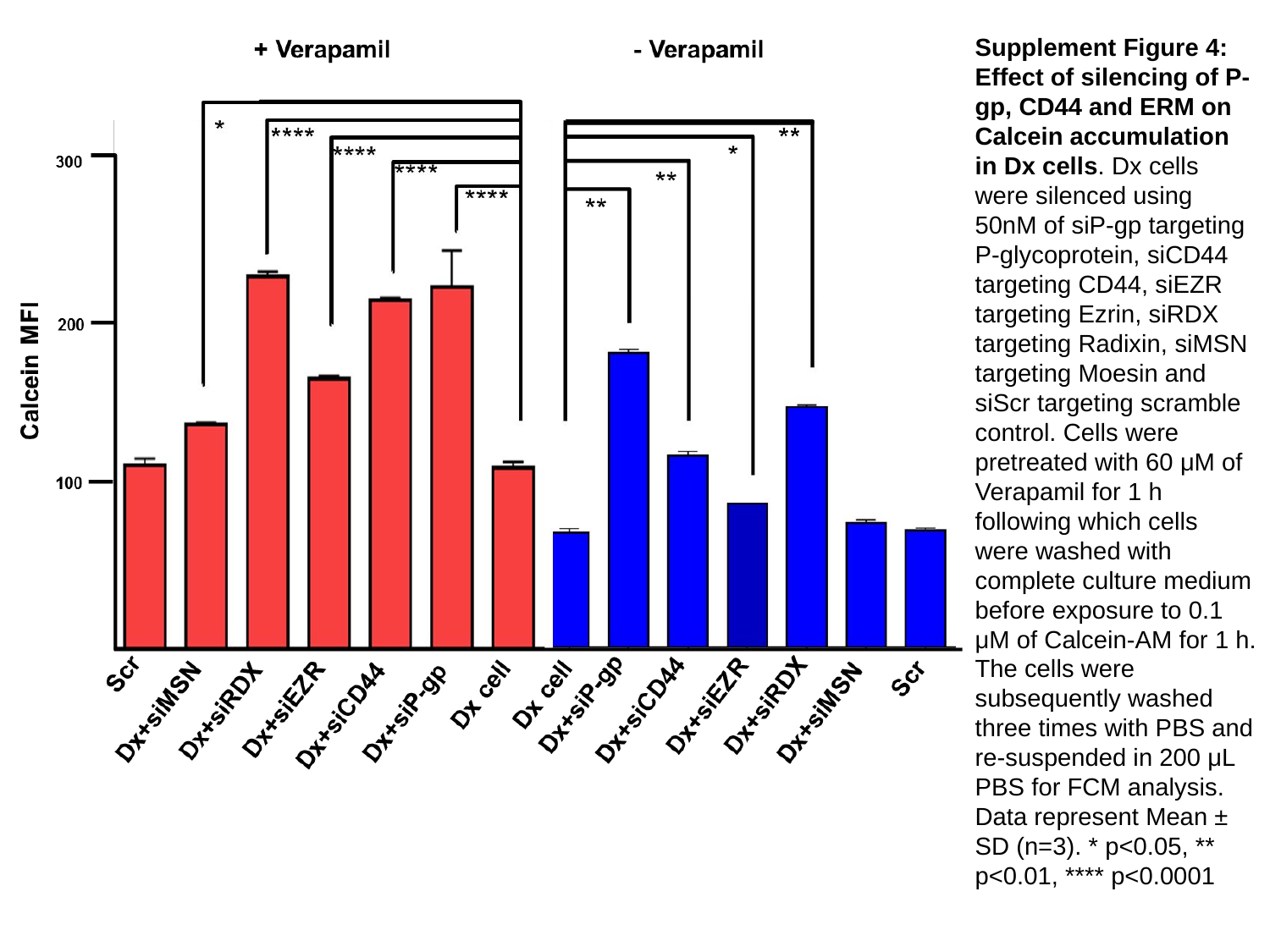

Supplement Figure 4: Effect of silencing of P-gp, CD44 and ERM on Calcein accumulation in Dx cells. Dx cells were silenced using 50nM of siP-gp targeting P-glycoprotein, siCD44 targeting CD44, siEZR targeting Ezrin, siRDX targeting Radixin, siMSN targeting Moesin and siScr targeting scramble control. Cells were pretreated with 60 μM of Verapamil for 1 h following which cells were washed with complete culture medium before exposure to 0.1 μM of Calcein-AM for 1 h. The cells were subsequently washed three times with PBS and re-suspended in 200 μL PBS for FCM analysis. Data represent Mean ± SD (n=3). * p<0.05, ** p<0.01, **** p<0.0001
